# Biochemical characterization of ClpB and DnaK from *Anaplasma phagocytophilum*

**DOI:** 10.1016/j.cstres.2024.06.003

**Published:** 2024-06-20

**Authors:** Chathurange B. Ranaweera, Sunitha Shiva, Swetha Madesh, Deepika Chauhan, Roman R. Ganta, Michal Zolkiewski

**Affiliations:** 1 Department of Biochemistry and Molecular Biophysics, Kansas State University, Manhattan, KS, USA; 2 Department of Diagnostic Medicine/Pathobiology, College of Veterinary Medicine, Kansas State University, Manhattan, KS, USA; 3 Department of Veterinary Pathobiology, College of Veterinary Medicine, University of Missouri, Columbia, MO, USA

**Keywords:** Molecular chaperone, ClpB, DnaK, Protein aggregation, *Anaplasma phagocytophilum*, Granulocytic anaplasmosis

## Abstract

*Anaplasma phagocytophilum* is an intracellular tick-transmitted bacterial pathogen that infects neutrophils in mammals and causes granulocytic anaplasmosis. In this study, we investigated the molecular chaperones ClpB and DnaK from *A. phagocytophilum*. In *Escherichia coli*, ClpB cooperates with DnaK and its co-chaperones DnaJ and GrpE in ATP-dependent reactivation of aggregated proteins. Since ClpB is not produced in metazoans, it is a promising target for developing antimicrobial therapies, which generates interest in studies on that chaperone’s role in pathogenic bacteria. We found that ClpB and DnaK are transcriptionally upregulated in *A. phagocytophilum* 3–5 days after infection of human HL-60 and tick ISE6 cells, which suggests an essential role of the chaperones in supporting the pathogen’s intracellular life cycle. Multiple sequence alignments show that *A. phagocytophilum* ClpB and DnaK contain all structural domains that were identified in their previously studied orthologs from other bacteria. Both *A. phagocytophilum* ClpB and DnaK display ATPase activity, which is consistent with their participation in the ATP-dependent protein disaggregation system. However, despite a significant sequence similarity between the chaperones from *A. phagocytophilum* and those from *E. coli*, the former were not as effective as their *E. coli* orthologs during reactivation of aggregated proteins *in vitro* and in supporting the survival of *E. coli* cells under heat stress. We conclude that the *A. phagocytophilum* chaperones might have evolved with distinct biochemical properties to maintain the integrity of pathogenic proteins under unique stress conditions of an intracellular environment of host cells.

## Introduction

*Anaplasma phagocytophilum* is a Gram-negative, obligate intracellular bacterium and one of the most clinically important tick-borne pathogens that cause infections in humans and several vertebrate hosts.^1^, ^2^ The *Anaplasma* genus is closely related, but distinct from the *Ehrlichia* genus within the Anaplasmataceae family in the order Rickettsiales.^1^ Human granulocytic anaplasmosis, an infection of neutrophils caused by *A. phagocytophilum* was identified only in the 1990s, and its etiology has not been adequately studied so far. Since human granulocytic anaplasmosis is a major public health concern, there is a growing interest in understanding the pathophysiology of *A. phagocytophilum* and its interactions with the host.

Pathogenic stress-response machinery, including molecular chaperones, emerged as an important factor that supports infectivity and in-host survival.^3^, ^4^, ^5^ We have previously shown that protein disaggregase ClpB from the AAA+ family of ATPases supports the proliferation of *Ehrlichia chaffeensis* during infection of mammalian cells by suppressing aggregation of bacterial proteins.^6^, ^7^ ClpB has been identified as an essential factor in multiple pathogenic microorganisms, including *Staphylococcus aureus,*^8^ *Mycobacterium tuberculosis,*^9^ *Plasmodium falciparum*,^10^ and other clinically important pathogens.^11^, ^12^ ClpB is produced in prokaryotes, lower eukaryotes (where it is known as Hsp100, Hsp101, or Hsp104), and plants, but, unlike other chaperones, it is not found in animals and humans.^13^ The apparent absence of ClpB in metazoans makes it an attractive target for developing novel antimicrobial therapies. Indeed, we have recently shown that targeting ClpB with a selective small-molecule ligand inhibits the growth of *Escherichia coli* and its survival under heat stress.^14^

ClpB reactivates aggregated proteins in collaboration with the DnaK/DnaJ/GrpE chaperone system.^15^ It has been shown that efficient cooperation of ClpB with DnaK occurs only for the chaperones from the same species, in spite of a high degree of sequence conservation among the ClpB and DnaK orthologs from different organisms.^15^, ^16^ The molecular mechanism and biological significance of developing a strong species-specificity for a protein quality control system are not fully understood. To advance understanding of the ClpB/DnaK bichaperone system in clinically important pathogens, we investigated the cooperation between ClpB and DnaK from *A. phagocytophilum* and the inter-species specificity with their chaperone orthologs from *E. coli*. We discovered that despite a significant sequence similarity, the *Anaplasma* chaperones cannot complement a lack of their native orthologs in *E. coli* cells.

## Results

### Analysis of the amino-acid sequence of *A. phagocytophilum* ClpB (ApB) and DnaK (ApK)

Supplementary Figures 1 and 2 include the sequence alignments of ClpB and DnaK from four bacterial species: *A. phagocytophilum*, its closely related rickettsial pathogen *E. chaffeensis*, *E. coli*, and *Thermus thermophilus*. High-resolution X-ray diffraction structural data are available for the *T. thermophilus* ClpB ortholog.^17^ Supplementary Figure 1 shows that *A. phagocytophilum* ClpB (ApB) contains all sequence regions that correspond to the domains identified in the structure of *T. thermophilus* ClpB: the N-terminal domain which supports ClpB binding to aggregated substrates,^18^ two nucleotide-binding domains, each containing the Walker A and B motifs, and the middle domain which couples ClpB with the DnaK system.^19^, ^20^, ^21^ The energy-supplying nucleotide-binding domains are the most conserved regions among the ClpB orthologs, while the N-terminal domain is the least conserved suggesting that substrate specificity of ClpB might differ for each of the bacterial species. However, the N-terminal domain of *A. phagocytophilum* ClpB does include three conserved residues that we have previously identified as essential for the disaggregase activity of *E. coli* ClpB: Thr7, Asp106, and Glu112.^22^

A unique feature of *E. coli* ClpB (EcB) is the efficient production of its N-terminally truncated isoform in addition to the full-length protein. The truncated ClpB isoform, which lacks the whole N-terminal domain, originates from an internal translation initiation site within the *E. coli* ClpB mRNA, which includes a secondary Shine–Dalgarno sequence.^23^ Importantly, the full-length and the truncated EcB isoforms synergize by forming hetero complexes that display a stronger disaggregation activity than each isoform separately.^24^, ^25^, ^26^ Sequence analysis of the *A. phagocytophilum* ClpB gene (not shown) did not reveal secondary Shine–Dalgarno motifs downstream from the initial ATG codon, which suggests that only the full-length protein is translated. Indeed, a recombinant expression of ApB produced a single major protein product, as resolved with SDS-PAGE (see Supplementary Figure 3).

ApK contains all the sequence regions found in its orthologs from other bacteria (Supplementary Figure 2): the nucleotide-binding domain, which also mediates the interactions with ClpB,^27^, ^28^ the substrate-binding domain, and the helical lid domain.^29^ All the structural domains of DnaK share strong sequence similarities among the four analyzed bacterial species, except the less conserved C-terminal tail of the lid domain. Overall, the sequence analysis suggests that ApB and ApK are *bona fide* orthologs of the chaperones from the previously studied bacteria and may participate in the protein aggregation control.

### Expression of ClpB and DnaK during the infectious cycle of *A. phagocytophilum*

**Fig. 1:**
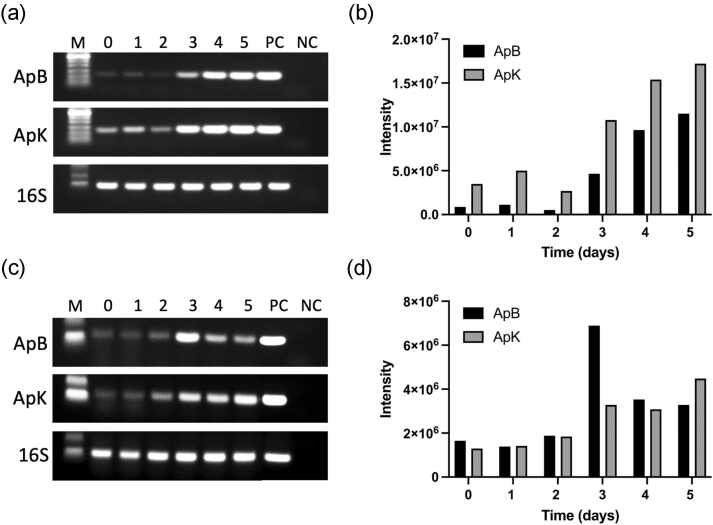
Expression of ClpB and DnaK during the infectious cycle of *A. phagocytophilum* in HL-60 cells (a and b) and in ISE6 tick cells (c and d). RNA samples recovered from infected *A. phagocytophilum* cultures over a period of 0–5 days were equalized after assessing their concentration by real-time PCR targeting 16S rRNA. Equal amounts of RNA from each time point were then used for determining the RNA expression levels for ApB and ApK by semi-quantitative RT-PCR. (a) and (c) represent agarose gel images of the resolved amplicons. (b) and (d) show the band intensities determined for each time point using the iBright image analysis software (Invitrogen). Lanes: M, molecular weight markers; numbers 0–5 refer to RNA recovered from the cultures at the days 0–5 post infection with cell-free *A. phagocytophilum*; PC, positive control where *A. phagocytophilum* genomic DNA was added as the template; NC, negative control reaction lacking a template DNA. Abbreviations used: ApB, ClpB from *A. phagocytophilum*; ApK, DnaK from *A. phagocytophilum*.

To determine how the expression of ClpB and DnaK is regulated in *A. phagocytophilum* upon infection of a mammalian and an invertebrate host, we investigated the chaperone transcript levels during infection of human promyeloblast cell line HL-60 (Figure 1(a) and (b)) and tick ISE6 cells (Figure 1(c) and (d)). ApB and ApK were strongly upregulated 3 days post-infection in human cell lines derived from blood lymphocytes (HL-60) and remained high during proliferation of the pathogen for up to 5 days (Figure 1(a) and (b)). In contrast, ApB expression in the tick cell line (ISE6) was upregulated transiently 3 days post-infection and returned to moderate levels in the later days of infection. The expression of ApK in ISE6 cells was upregulated 3 days post-infection and remained elevated for up to 5 days (Figure 1(c) and (d)).

### Biochemical properties of *A. phagocytophilum* ClpB and DnaK

**Fig. 2:**
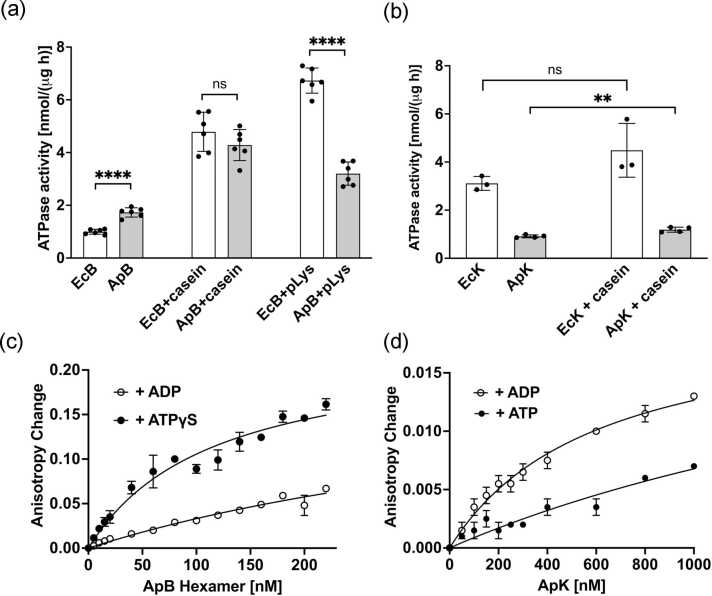
Biochemical properties of the recombinant *A. phagocytophilum* ClpB and DnaK. (a) The ATPase activity of ClpB from *E. coli* (EcB) and *A. phagocytophilum* (ApB). (b) The ATPase activity of DnaK from *E. coli* (EcK) and *A. phagocytophilum* (ApK). The ATPase activity was measured at 37 °C in the absence of other factors (basal activity), in the presence of 0.1 mg/mL κ-casein, or 0.03 mg/mL poly-lysine. The average values from multiple measurements are shown with the standard deviations. Unpaired t-test analysis was performed with GraphPad Prism software (****P < 0.0001; **P < 0.01, ns, *P* > 0.05). (c and d) Fluorescence anisotropy changes observed upon interaction between ApB (c) and ApK (d) with the FITC-labeled peptide B2 in the presence of 2 mM ADP, ATP, or ATPγS. The average values from two experiments are shown with the standard deviations. The solid lines indicate fits of the hyperbolic binding isotherms using GraphPad Prism software. Abbreviations used: pLys, poly-lysine; FITC, fluorescein isothiocyanate; ATPγS, adenosine 5'-(3-thiotriphosphate).

We produced and purified recombinant ApB and ApK using an *E. coli* expression system (Supplementary Figure 3). As shown in Figure 2(a), the basal ATPase activity of ApB exceeded that of EcB. It was previously shown that the ATPase activity of EcB increased in the presence of pseudo-substrates, casein, and poly-lysine.^22^, ^30^ EcB and ApB showed similar ATPase activities in the presence of casein, while the response to poly-lysine was less pronounced for ApB, compared to EcB (Figure 2(a)). The basal ATPase activity of ApK was lower than that of EcK (Figure 2(b)). The ATPase of ApK increased slightly in the presence of casein, whereas the ATPase of EcK did not respond to casein (Figure 2(b)).

Both ClpB and DnaK use energy from ATP hydrolysis to induce conformational changes in their protein substrates.^15^ However, each chaperone displays a unique preference for nucleotide occupancy to induce high-affinity substrate binding. While the ATP-bound state of ClpB shows strong binding to substrates,^31^ it is the ADP-bound state that stabilizes DnaK complexes with its substrate proteins.^32^ We investigated if the *A. phagocytophilum* orthologs of ClpB and DnaK conform to the above paradigm. We used fluorescence anisotropy to obtain binding isotherms for the chaperones’ interaction with a model peptide B2 that binds to both EcB and EcK.^33^, ^34^ As shown in Figure 2(c), the binding of fluorescein isothiocyanate (FITC)-labeled B2 to ApB was stronger in the presence of a non-hydrolyzable ATP analog, adenosine 5'-(3-thiotriphosphate) (ATPγS), than in the presence of ADP, which agrees with the nucleotide-preference of EcB. In contrast to ApB, and as expected for DnaK orthologs, ADP induced a strong binding of B2 to ApK (Figure 2(d)).

### ClpB/DnaK-mediated aggregate reactivation *in vitro*

**Fig. 3:**
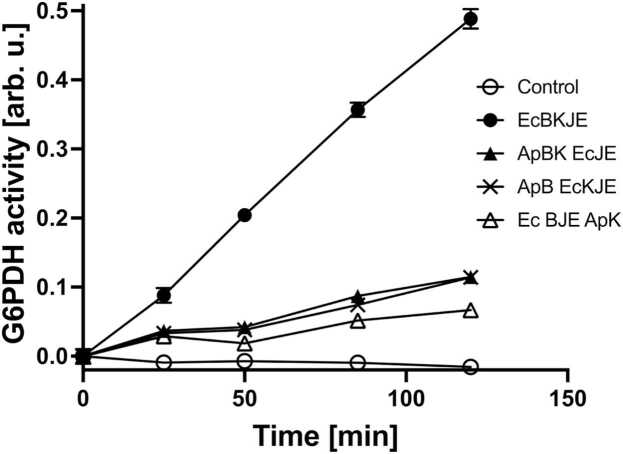
Aggregate reactivation activity of the ClpB-DnaK bichaperone system. Aggregated glucose-6-phosphate dehydrogenase was incubated without chaperones (Control) or with the indicated sets of the chaperones ClpB (B), DnaK (K), DnaJ (J), and GrpE (E) from *E. coli* (Ec) or *A. phagocytophilum* (Ap) and its enzymatic activity was determined at the indicated time. Shown are the average values from three measurements with the standard deviations.

We have previously used bacterial glucose-6-phosphate dehydrogenase (G6PDH) as a model substrate in aggregate-reactivation assays.^14^, ^26^, ^35^ G6PDH is a dimeric enzyme that does not spontaneously refold after urea-induced unfolding and whose reactivation strictly depends on the ClpB/DnaK activity. Indeed, as shown in Figure 3, a time-dependent recovery of G6PDH activity was observed in the presence of ClpB and the DnaK system (DnaK/DnaJ/GrpE) but not without the chaperones. Notably, the rate of G6PDH reactivation was lower for ApB and ApK when paired with *E. coli* DnaJ and GrpE (ApBK EcJE) than for the reactivation mediated by the full set of *E. coli* chaperones (EcBKJE). Also, the rate of G6PDH reactivation mediated by the *E. coli* chaperones was low when EcK was replaced with the *A. phagocytophilum* ortholog (EcBJE ApK) or when EcB was replaced with ApB (ApB EcKJE). The results in Figure 3 reveal that while *A. phagocytophilum* ClpB and DnaK did show aggregate reactivation activity, but their cooperation with *E. coli* co-chaperones was suboptimal.

### Replacement of the *E. coli* chaperones with *A. phagocytophilum* ClpB and DnaK in bacterial cells

**Fig. 4:**
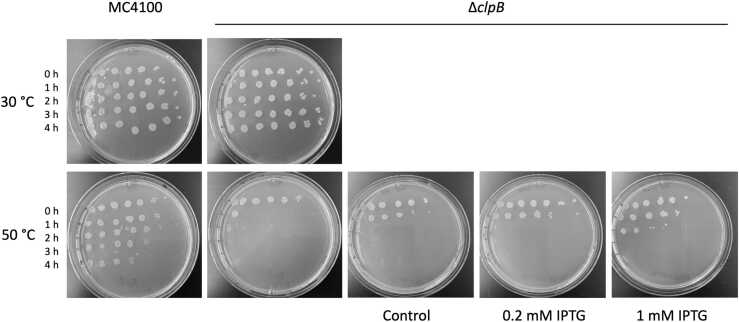
Viability of *E. coli* and its ClpB-deficient strain under heat shock and upon expression of *A. phagocytophilum* ClpB. The indicated strains of *E. coli* were incubated at the indicated temperature, then spotted on agar plates, and incubated overnight at 37 °C. Each spot on the agar plates represents a viable culture after a 10-fold serial dilution (from left to right). Each row on the plates corresponds to a different time of incubation at a given temperature in hours. For expression of ApB, the *E. coli* Δ*clpB* strain was transformed with pCBR_AphClpB (see Materials and methods) and pCS6 for production of T7 RNA polymerase. Production of ApB was induced with the indicated concentration of IPTG and confirmed with western blotting using polyclonal antisera raised against recombinant ClpB from *Ehrlichia chaffeensis* (Supplementary Figure 3). Shown are representative results from three repeated experiments. Abbreviations used: ApB, ClpB from *A. phagocytophilum*; IPTG, isopropyl ß-D-1-thiogalactopyranoside.

We asked if ApB can functionally complement a lack of the native ClpB in *E. coli* cells. As shown in Figure 4, the wild-type *E. coli* strain survived up to 4 h under severe heat shock at 50 °C, but the bacterial viability was rapidly lost at that temperature in the strain that did not produce ClpB (Δ*clpB*). Expression of ApB in the Δ*clpB* background produced only a modest viability enhancement (Figure 4, 1 mM isopropyl ß-D-1-thiogalactopyranoside (IPTG) condition, and Supplementary Figure 4). Thus, ApB cannot rescue the heat-sensitive phenotype of the Δ*clpB* strain.

**Fig. 5:**
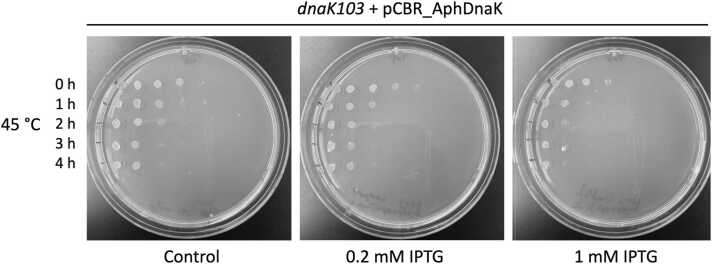
Viability of *E. coli dnaK103* strain upon expression of *A. phagocytophilum* DnaK. *E. coli* cultures were incubated at 45 °C, then spotted on agar plates, and incubated overnight at 37 °C. Each spot on the agar plates represents a viable culture after a 10-fold serial dilution (from left to right). Each row on the plates corresponds to a different time of incubation at 45 °C in hours. For expression of ApK, the *dnaK103* strain was transformed with pCBR_AphDnaK (see Materials and methods) and pCS6 for production of T7 RNA polymerase. Production of ApK was induced with the indicated concentration of IPTG and confirmed with western blotting using polyclonal antisera raised against recombinant DnaK from *Ehrlichia chaffeensis* (Supplementary Figure 3). Shown are representative results from three repeated experiments. Abbreviations used: ApK, DnaK from *A. phagocytophilum*; IPTG, isopropyl ß-D-1-thiogalactopyranoside.

To determine if ApK can replace EcK *in vivo*, we employed an *E. coli* strain *dnaK103*, that produces a truncated inactive DnaK. The DnaK variant in the *dnaK103* strain does not bind to its substrates because it contains an incomplete substrate-binding domain, which significantly inhibits bacterial heat tolerance.^36^ As shown in Figure 5, the *dnaK103* strain poorly survived a moderate heat shock at 45 °C, and its viability was not rescued upon expression of ApK (Figure 5 and Supplementary Figure 4).

**Fig. 6:**
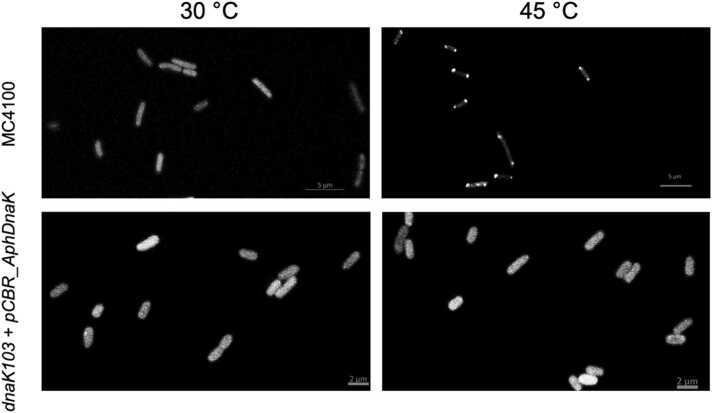
Localization of ClpB-YFP in *E. coli* under heat stress and upon expression of *A. phagocytophilum* DnaK in the DnaK-deficient background. The indicated strains of *E. coli* were grown at the indicated temperatures and the localization of ClpB-YFP was determined with confocal microscopy. For expression of ApK, the *dnaK103* strain was transformed with pCBR_AphDnaK (see Materials and methods) and pCS6 for production of T7 RNA polymerase. Production of ApK was induced with 0.2 mM IPTG. Abbreviations used: ApK, DnaK from *A. phagocytophilum*; IPTG, isopropyl ß-D-1-thiogalactopyranoside; ClpB-YFP, ClpB fused with yellow fluorescent protein.

It has been shown that heat-induced aggregates of thermo-sensitive proteins accumulate at the poles of *E. coli* cells.^37^ Importantly, DnaK and ClpB colocalize with the aggregates, but an absence of a functional DnaK prevents ClpB from accumulating with the aggregates.^14^, ^38^ As shown in Figure 6 (upper panels), ClpB fused withyellow fluorescent protein (YFP) accumulates at the cell poles under heat-shock conditions, in agreement with the previous results. However, the pole accumulation of ClpB was not observed in the *dnaK103* strain (Figure 6, lower panels), even upon expression of the full-length ApK (see Supplementary Figure 4). Thus, ApK cannot functionally complement a lack of the native DnaK in *E. coli* cells in targeting of ClpB to protein aggregates.

## Discussion

We performed the first biochemical characterization of the bichaperone system, ClpB/DnaK, from the tick-transmitted intracellular bacterial pathogen *A. phagocytophilum*. Analysis of the amino-acid sequence of both chaperones demonstrates strong conservation of protein domains and essential sequence motifs between *A. phagocytophilum* ClpB and DnaK and the corresponding sequences from several previously studied bacterial species (Supplementary Figures 1 and 2). Previous studies on the bichaperone system from *E. coli* showed that ClpB and DnaK cooperate during the reactivation of aggregated proteins and support *E. coli* survival under heat stress.^39^, ^40^ Further research demonstrated that ClpB is essential for the survival and virulence of many bacterial pathogens.^12^ Since there are no apparent orthologs of ClpB in metazoans, that chaperone became a promising target for antimicrobial treatments.^14^, ^41^ The sequence similarity between ClpB and DnaK from *A. phagocytophilum* and from other bacteria (Supplementary Figures 1 and 2) suggests that those proteins may control the protein aggregation in *A. phagocytophilum* and, thus, may be targeted to attenuate proliferation of the pathogen.

The importance of the ClpB/DnaK system in the life cycle of *A. phagocytophilum* is indicated by transcriptional upregulation of both chaperones during the pathogen proliferation inside the infected human and tick cells. The mRNA levels of ApB and ApK are elevated during the most intense phase of *A. phagocytophilum* replication until the pathogen is released from infected cells in the form of dense-core particles which can reinfect other cells^42^ (Figure 1(a) and (b)). In contrast to HL-60 infection, the ApB mRNA is transiently upregulated after infection of ISE6 cells (Figure 1(c) and (d)), which mirrors the ClpB expression pattern during the infection of macrophages with an intracellular pathogen *E. chaffeensis.*^6^ Altogether, the results in Figure 1 suggest that *A. phagocytophilum* ClpB and DnaK play a role in the pathogen’s adjustment to an intracellular environment of the host and the in-host proliferation.

The biochemical properties of recombinant ApB and ApK agree with those of their previously studied orthologs (Figure 2). The ATPase activity of ApB is upregulated in the presence of pseudo-substrates, casein and poly-lysine, which mirrors the known propensity of EcB to interact with poorly soluble and/or positively charged polypeptides.^22^, ^33^ Notably, ApB and ApK reproduce the known property of ClpB and DnaK of modulating their affinity toward substrates in response to nucleotide binding (Figure 2(c) and (d)).

Lower rates of reactivation of the aggregated G6PDH were observed with the chaperone sets composed of both *A. phagocytophilum* and *E. coli* proteins, as compared to the activity of an all-*E. coli* chaperone system (Figure 3) recapitulate the previously observed apparent “species specificity” of the ClpB/DnaK system.^16^, ^43^, ^44^ The experiments shown in Fig. 3, Fig. 4, Fig. 5, Fig. 6 suggest that *A. phagocytophilum* ClpB and DnaK do not cooperate efficiently with their *E. coli* chaperone partners *in vivo* and *in vitro*. A suboptimal level of cooperation with *E. coli* chaperones was also previously observed for ClpB from *E. chaffeensis.*^6^ Several factors could potentially contribute to the suboptimal activity of ApB and ApK as compared to the *E. coli* chaperones *in vitro* and in the environment of *E. coli* cells. First, despite the overall sequence similarity between ClpB and DnaK from different bacterial species (Supplementary Figures 1 and 2), the motifs that mediate interactions between the co-chaperones may not be conserved. In *E. coli* ClpB, previous studies identified the C-terminal region of the middle domain (Ser456-Glu520) as the site of interaction with DnaK.^21^ Within the middle domain of ClpB from *T. thermophilus*, Tyr484 and Tyr494 are essential for interaction with DnaK and participate in a network of intermolecular hydrogen bonds.^27^ The former residue is replaced with Gly in ApB, while the latter residue is conserved in both ClpB orthologs (Supplementary Figure 1). On the DnaK side of the interface, multiple residues within the nucleotide-binding domain were found essential for interaction with ClpB.^28^ Those residues include a few that are conserved in ApK and several that are not conserved: Val59 in EcK is replaced with Asn in ApK, Thr60 with Ile, Leu257 with Met, Asn282 with Thr, and Tyr285 with Phe. A replacement of several key residues in ClpB and DnaK within the interaction interface could disturb the network of hydrogen bonds and hydrophobic interactions that stabilize the bichaperone complex^27^ and could contribute to suboptimal cooperation between the chaperones from *A. phagocytophilum* and *E. coli*.

Moreover, the incompatibility of the nucleotide-exchange factor GrpE from *E. coli* with the bichaperone system from *A. phagocytophilum* could also contribute to its low efficiency in aggregate reactivation. It has been shown that ClpB and GrpE compete for the interaction site in DnaK and that a strong GrpE binding to DnaK inhibits the ClpB-mediated protein disaggregation.^27^ Notably, while the sequence identity between ApB and EcB is 53 %, between ApK and EcK is 60 %, and between the corresponding DnaJ orthologs is 48 %, the identity between *A. phagocytophilum* and *E. coli* GrpE is only 28 %, which highlights a potential role of the nucleotide-exchange factor in the apparent species specificity of the bichaperone systems. Future studies on GrpE from *A. phagocytophilum* may determine if the differences in affinity of GrpE toward different DnaK orthologs correlate with the aggregate reactivation activity of the ClpB/DnaK chaperone system.

Finally, it should be noted that the multichaperone systems in different bacteria evolved in response to distinct types of environmental stresses. In contrast to *E. coli*, replication of *A. phagocytophilum* requires an intracellular environment of a mammalian or invertebrate host. In mammals, *A. phagocytophilum* is maintained predominantly in mature neutrophils within distinct membrane-enclosed phagocytized vacuoles (referred to as morulae) where the bacterium replicates.^2^ The *A. phagocytophilum* morulae show characteristics of autophagosomes but do not fuse with lysosomes. Proliferation inside the inclusions allows *A. phagocytophilum* to avoid the neutrophil antimicrobial machinery but possibly exposes the pathogen to a different level and duration of environmental stress than typically experienced by extracellular microorganisms, like *E. coli*. These stresses include competing with the neutrophil host in acquiring the essential metabolites, minerals, and nutrients. As a result, the identity, structural properties, and sensitivity to aggregation/disaggregation of the potential ClpB/DnaK client proteins may differ in *A. phagocytophilum* and in other bacteria. Thus, one can speculate that the *A. phagocytophilum* and *E. coli* chaperones divergently evolved to rescue different sets of protein clients. Indeed, the sequence conservation between ClpB from *A. phagocytophilum* and *E. coli* is weak within the N-terminal domain, which mediates interactions with aggregated substrates^18^ (Supplementary Figure 1). In addition, *E. coli* ClpB optimizes its protein disaggregating activity *via* hetero-association of its full-length and truncated isoforms, which enhances the mobility of the substrate binding N-terminal domain.^26^ In contrast to *E. coli*, the N-terminally truncated isoform of ClpB is not produced in *A. phagocytophilum* (Supplementary Figure 3), which suggests that the N-terminal domain’s mobility might be more restricted in *A. phagocytophilum* ClpB than in *E. coli* ClpB.

## Conclusion

The intracellular tick-borne bacterial pathogen *A. phagocytophilum* upregulates the production of the chaperones ClpB and DnaK during the infection of mammalian and invertebrate cells. Despite a significant sequence similarity between ClpB and DnaK from *A. phagocytophilum* and their orthologs from other bacteria, the *Anaplasma* chaperones cannot functionally complement a lack of the native ClpB or DnaK to support the survival of *E. coli* cells under heat stress. These studies suggest that chaperones in intracellular pathogens might have evolved unique biochemical properties to maintain the integrity of pathogenic proteomes under specific conditions of in-host proliferation.

## Materials and methods

### DNA constructs

The protein-coding sequences of *A. phagocytophilum* ClpB and DnaK were PCR amplified using bacterial genomic DNA isolated with DNeasy blood and tissue kit (Qiagen Sciences Inc, Germantown, MD) as the template and the respective gene-specific primers designed for targeted cloning into the *E. coli* expression plasmid vector, pET-28a(+) (Novagen/Millipore-Sigma, Burlington, MA). The primers used to produce the *A. phagocytophilum* ClpB and DnaK constructs are listed in Supplementary Table 1. The ClpB and DnaK PCR products were digested with *Nhe*I and *Xho*I and cloned into pET-28a(+) vector similarly digested with the above restriction enzymes. The resulting plasmids pCBR_AphClpB and pCBR_AphDnaK were propagated in NEB 5-alpha *E. coli* cells (New England Biolabs, Ipswich, MA). For protein production, pCBR_AphClpB and pCBR_AphDnaK plasmids were transformed into *E. coli* BL21(DE3) cells (Novagen/Millipore-Sigma).

### Proteins

*E. coli* BL21(DE3) cells carrying pCBR_AphClpB or pCBR_AphDnaK were spread on agar plates supplemented with kanamycin (50 μg/mL) and incubated overnight at 37 °C. A single colony carrying each recombinant plasmid was used to inoculate a fresh Luria-Bertani (LB) media supplemented with kanamycin, followed by incubation at 37 °C overnight. The final culture was used to prepare glycerol stocks for further use. To produce recombinant ApB and ApK, a previously prepared glycerol stock was used to inoculate LB media supplemented with kanamycin (50 μg/mL). After overnight incubation at 30 °C, the culture was diluted 50-fold in 1 L of LB with kanamycin and incubated at 30 °C. When absorbance at 600 nm reached approximately 0.4, IPTG was added to a ﬁnal concentration of 1 mM, followed by the culture incubation overnight at 20 °C with shaking at 250 RPM. Cells were collected by centrifugation (Beckman JA-14 rotor, 3000 g relative centrifugal force, 15 min, 4 °C). The cell pellet was resuspended in 20 mL of cold Ni-NTA lysis buffer (40 mM Tris-HCl, pH 8.0, 100 mM KCl, 10 mM imidazole) by vortexing. Phenylmethylsulfonyl fluoride was added to a final concentration of 1 mM together with 1 tablet of cOmpleteTM Protease Inhibitor (Promega, Madison, WI) per 50 mL of sample. The cells were disrupted by sonication on ice using pre-chilled tubes. The soluble fraction was collected by centrifugation (Beckman JA-20 rotor, 11,500 RPM, 60 min, 4 °C). The supernatant was loaded onto a column with 5 mL of Ni-NTA resin (Qiagen) equilibrated with Ni-NTA lysis buffer. The column was washed with 50 mL of Ni-NTA wash buffer (40 mM Tris-HCl, pH 8.0, 100 mM KCl, 20 mM imidazole). Proteins were eluted in 5-mL fractions with Ni-NTA elution buffer (40 mM Tris-HCl, pH 8.0, 100 mM KCl, 250 mM imidazole). The fractions containing either ApB or ApK were pooled and concentrated with a centrifugal ﬁlter device (10,000 Da molecular weight cut-off, 3500 RPM, 4 °C). The concentrated ApB and ApK were further subjected to gel filtration chromatography on a Superdex 200 column equilibrated with the gel filtration buffer (50 mM Tris-HCl, pH 7.5, 0.2 M KCl, 20 mM MgCl_2_, 10 % glycerol, 1 mM EDTA, 1 mM DTT). The fractions containing purified proteins with purity greater than 95 % were pooled and subjected to thrombin (Sigma-Aldrich, St. Louis, MO) to remove the N-terminal 6-His tag. The purity of the thrombin-cleaved samples was assessed with SDS-PAGE and the final protein concentration was measured using the Bradford method. Aliquots of the purified recombinant ApB and ApK were stored at −20 °C in the gel filtration buffer.

G6PDH from *Leuconostoc mesenteroides* was obtained from Sigma-Aldrich. *E. coli* ClpB was produced and purified as described before.^45^ *E. coli* DnaK, DnaJ, and GrpE were obtained from Enzo Life Sciences (Farmingdale, NY). Protein concentrations were determined spectrophotometrically and are reported as monomer concentrations. Peptide B2 was purchased from Peptide 2.0 (Chantilly, VA). The peptide was N-terminally labeled with fluorescein isothiocyanate (FITC) and C-terminally amidated by the manufacturer. The peptide concentration was determined spectrophotometrically using the FITC molar extinction coefficient 77,000 cm^−1^ M^−1^ at 494 nm.

### Bacterial strains

The following strains of *E. coli* were used in the experiments shown in Fig. 2, Fig. 3, Fig. 4: MC4100, MC4100Δ*clpB:kan,*^23^ and *dnaK*103.^36^

### *In vitro* cultivation of *A. phagocytophilum*

*A. phagocytophilum* HGE2 isolate^46^ was used to infect the human promyelocytic cell line; HL-60 (ATCC CCL-240, Manassas, VA). *A. phagocytophilum* in HL-60 was cultured as previously described.^47^ To maintain *A. phagocytophilum*, 100 µL of the pathogen-infected precultured HL-60 cells were added to 5 mL media containing 1 × 10^5^ HL-60 cells in a 25-cm^2^ flask. *A. phagocytophilum* was propagated in ISE6 tick cells as described previously.^48^ A monolayer of ISE6 cells in a 25-cm^2^ flask (∼3 × 10^6^) was infected with 100 µL of the previously infected tick cells showing ∼90 % infectivity.

To investigate the gene expression levels of *A. phagocytophilum* in HL-60 and ISE6 cells, host cell-free bacteria were isolated as described previously.^49^ Briefly, ∼90 % infected HL-60 cells (∼5 × 10^6^ cells) were harvested by centrifugation at 15,000 *g* for 15 min at 4 °C. The supernatant was discarded, and the pellet was resuspended in 1.5 mL of ice-cold 0.3 M sucrose solution to which 0.1 mL of silicone carbide (Lortone Inc., Mukilteo, WA) was added and cells and lysed by vortexing for 30 s, followed by centrifugation at 200 *g* for 5 min at 4 °C to separate the cell debris. The supernatant was then passed through a 2 µm filter (Whatman Ltd., Clifton, NJ) and centrifuged at 15,000 *g* for 10 min at 4 °C. The pellet containing the host cell-free bacteria was resuspended in 180 µL 0.3 M sucrose solution and added to ∼12 × 10^6^ HL-60 cells in 3 mL culture media. Cells were incubated at 37 °C for an hour with gentle mixing once every 15 min. Cells were then collected by centrifugation at 350 *g* for 6 min, media was discarded, and the pellet was resuspended in 5 mL culture media and centrifuged again to remove any unbound bacteria. Subsequently, the cell pellet was resuspended in 120 mL of culture media at 37 °C and 20 mL each was transferred to six 25-cm^2^ flasks and incubated at 37 °C until harvested. The cultured cells were harvested from one flask each at days 0–5 and stored at −80 °C in Trizol solution for RNA isolation.

Cell-free *A. phagocytophilum* isolated from ISE6 cells from ∼90 % infected 25-cm^2^ flask (∼3 × 10^6^ cells) was also used for the time-course experiment. The cell-free *A. phagocytophilum* was resuspended in 180 µL of 0.3 M ice-cold sucrose solution. Thirty microliter each of the purified bacteria was added to six 25-cm^2^ flasks containing ∼3 × 10^6^ ISE6 cells (∼80 % confluency). The infected flasks were incubated at 34 °C and gently swirled once every 15 min. After an hour, media were removed, and fresh 5 mL of infection media was added to each flask, swirled, and discarded. Subsequently, 5 mL of infection media per flask was added, and the incubation was continued for 0–5 days. Cells were harvested from each flask by centrifugation at 15,000 *g* for 15 min at 4 °C, and the cell pellets were stored in 1 mL Trizol at −80 °C for RNA isolation.

### Determination of *A. phagocytophilum* gene expression levels

*A. phagocytophilum* RNA was recovered from the infected HL-60 and ISE6 cultures over a period of 0–5 days as described above and its concentration was estimated by real-time PCR targeted to 16S rRNA.^50^ Equal amounts of RNA from each time point were used to determine the expression levels for ApB, ApK, and 16S rRNA with gene-specific primers (Supplementary Table 2) by semi-quantitative RT-PCR (30 cycles for RNA derived from HL-60, 35 cycles for RNA from ISE6) as previously described.^6^ PCR products were resolved on a 1 % agarose gel. Band intensities of the specific amplicons for different time points were determined with iBright image analysis software (Invitrogen, Carlsbad, CA).

### Bacterial viability

*E. coli* MC4100, MC4100Δ*clpB*, and *dnaK103* strains were maintained in LB media, and in the case of the MC4100Δ*clpB* strain, LB media were supplemented with 30 μg/mL kanamycin. All experiments were initiated by preparing overnight cultures inoculated from single colonies and grown at 37 °C. The *dnaK103* cultures were grown at 30 °C. On the following day, the cultures were diluted 100-fold in 10 mL of LB media without antibiotics and incubated at 37 °C. When the culture absorbance reached ∼0.4 at 600 nm, the samples were transferred into an incubator set at 30, 45, or 50 °C. Bacteria were cultured for up to 4 h with shaking (200 RPM). At speciﬁc time points (see the legends to Fig. 4, Fig. 5), 100 µL of each culture was withdrawn, serially diluted in sterile 0.9 % NaCl up to 10^6^-fold dilution and spotted on LB agar plates. After complete adsorption of liquid on the LB agar surface, the plates were incubated overnight at 37 °C.

### Confocal microscopy

ClpB fused with yellow fluorescent protein (ClpB-YFP) was expressed at low levels in *E. coli* strains Δ*clpB*, and *dnaK*103 as described before.^37^ The expression of ClpB-YFP was initiated by the addition of 200 μM IPTG. The bacterial cells were cultured at 30 °C. Protein translation was stopped by the addition of erythromycin (30 μg/mL). Confocal imaging was performed as described previously.^14^

### Western blotting

*A. phagocytophilum* ClpB and DnaK were detected using the polyclonal rabbit antisera raised against a recombinant *E. chaffeensis* ClpB^6^ and DnaK by Thermo Fisher Scientific commercial services. Secondary anti-rabbit antibody conjugated with horseradish peroxidase (Sigma-Aldrich) and SuperSignal™ West Pico Chemiluminescent Substrate (Thermo Fisher Scientific) were used for the signal detection.

### ATPase activity assays

ClpB aliquots (1.8 µg) were diluted in 19 µL of buffer C (50 mM Tris-HCl pH 7.4, 20 mM MgCl_2_ 1 mM EDTA, 0.5 mM tris(2-carboxyethyl)phosphine, TCEP) in a 96-well plate. In some experiments, the buffer was supplemented with 0.1 mg/mL κ-casein or 0.03 mg/mL poly-lysine (Sigma-Aldrich). After a 10-min pre-incubation at 37 °C, the ATP hydrolysis reaction was initiated by adding 1 µL of 100 mM ATP. The samples were incubated for 60 min (basal ClpB activity) or 20 min (in the presence of κ-casein or poly-lysine) at 37 °C. After incubation, 200 µL of the ammonium molybdate/malachite green reagent^51^ was added to the samples on the 96-well plate, followed by the addition of 30 µL of 34 % sodium citrate.^52^ The plate was agitated inside a Synergy H1 reader (BioTek, Winooski, VT) for 15 min at room temperature, and the absorbance was measured at 630 nm. The readouts for ClpB-containing samples were corrected for absorbance of samples without ClpB, to account for non-enzymatic production of inorganic phosphate. A standard curve obtained with different inorganic phosphate concentrations was used to determine the amount of phosphate produced from ATP in the presence of ClpB.

To determine the ATPase activity of DnaK, 2.4 µg of DnaK was diluted in 19 µL of buffer C in a 96-well plate. Samples without DnaK were used as a control. The samples were pre-incubated for 10 min at 37 °C. The ATP-hydrolysis reaction was initiated by adding 1 µL of 100 mM ATP. The samples were incubated at 37 °C for 60 min (basal activity) or 30 min (in the presence of κ-casein). The concentration of inorganic phosphate produced from ATP in the presence of DnaK was determined as described above for ClpB.

### Fluorescence anisotropy

Fluorescence anisotropy measurements were performed using a PerkinElmer LS55 fluorescence spectrometer equipped with automatic polarizers, as described before.^34^

### Aggregate reactivation assays

To denature G6PDH, 5 µL of the G6PDH stock solution (441 μM) was mixed with 5 µL of buffer A (10 M urea, 16 % glycerol, 40 mM DTT) and incubated for 5 min at 47 °C. Subsequently, 90 µL of buffer B (50 mM Tris-HCl, pH 7.4, 20 mM Mg(Oac)_2_, 30 mM KCl, 1 mM EDTA, 1 mM β-mercaptoethanol) preheated at 47 °C was added to initiate G6PDH refolding, which results in strong protein aggregation.^53^ G6PDH aggregate production was arrested after 15 min by transferring into ice for 2 min. To observe aggregate reactivation, aggregated G6PDH was diluted 10-fold in buffer C (50 mM Tris-HCl, pH 7.4, 20 mM MgCl_2_, 1 mM EDTA, 0.5 mM TCEP) containing the chaperones: 5.5 μM DnaK, 1.3 μM DnaJ, 1.1 μM GrpE, 9 μM ClpB. Aggregated G6PDH in buffer C without chaperones was used as a control. The samples were incubated at 30 °C for the time indicated in Figure 6. Then, a 10-µL aliquot was mixed with 190 µL of buffer D (50 mM Tris-HCl, pH 7.8, 5 mM MgCl_2_, 1.5 mM glucose-6-phosphate, 1 mM NADP^+^) in a 96-well plate that had been preheated at 30 °C. The plate was inserted into the Synergy H1 plate reader (BioTek) with the chamber temperature at 30 °C, and the sample absorbance at 340 nm was measured after 10 min.

## Author contributions

Chathurange B. Ranaweera: Investigation, Data Curation, Writing – Review & Editing. Sunitha Shiva: Investigation, Data Curation. Swetha Madesh: Investigation, Data Curation, Writing – Review & Editing. Deepika Chauhan: Investigation, Data Curation, Writing – Review & Editing. Roman R. Ganta: Conceptualization, Methodology, Supervision, Funding acquisition, Writing – Review & Editing. Michal Zolkiewski: Conceptualization, Methodology, Supervision, Funding acquisition, Writing – Original Draft.

## Declarations of interest

The authors declare that they have no known competing financial interests or personal relationships that could have appeared to influence the work reported in this paper.

## Data Availability

Data will be made available on request.
