# Supplementary material for: Biochemical characterization of ClpB and DnaK from Anaplasma phagocytophilum
Source: Cell Stress Chaperones. 2024 Jun 20;29(4):540–51. doi: 10.1016/j.cstres.2024.06.003 (PMC11268196; doi:10.1016/j.cstres.2024.06.003)

Supplementary material

Biochemical characterization of ClpB and DnaK from *Anaplasma phagocytophilum*

Chathurange B. Ranaweera, Sunitha Shiva, Swetha Madesh, Deepika Chauhan, Roman R. Ganta, and Michal Zolkiewski

Supplementary Tables 1 – 2
Supplementary Figures 1 – 4

Supplementary Table 1

Oligonucleotide primers used to produce the *A. phagocytophilum* ClpB and DnaK constructs. The restriction sites for NheI and XhoI are underlined.

Target gene	Primer name	Sequence
**ApB**	ClpB-NheI	5'-ACTGAGCTAGCATGGACTTGAATAAGTTTACTGA-3'
	ClpB-XhoI	5'-TCAGTCTCGAGTTATGTAGCCTCTTTGATAACTAG-3'
**ApK**	DnaK-NheI	5'-ACTGAGCTAGCATGGGGGTAGTCATGG-3'
	DnaK-XhoI	5'-TCAGTCTCGAGCTAAGTATTCTTCTTGTCCTCG-3'

Supplementary Table 2

Oligonucleotide primers used in RT-PCR.

Target gene	Primer name	Sequence
**ApB**	ClpB-Forward	5'-GACCCTGTAATCGGTAGAAG-3'
	ClpB-Reverse	5'-GGGGACATTTCCGGAAAC-3'
**ApK**	DnaK-Forward	5'-GGTGACAAGCAAAGGACT-3'
	DnaK-Reverse	5'-CATCATATGTTCCATGATGGC-3'
**16S rRNA**	16SrRNA-Forward	5'-CCTACAGAAGAAGTCCCG-3'
	16SrRNA-Reverse	5'-GTTAAGCCCTGGCATTTC-3'
	16S rRNA probe	5'-TET/TTGCTATAAAGAATAATTAGTGGCAGACG/DABCYL-3'

Supplementary Figure 1

Sequence alignment of ClpB from *Anaplasma phagocytophilum*, *Ehrlichia chaffeensis*, *Escherichia coli*, and *Thermus thermophilus*. The alignment was produced with ClustalOmega (https://www.uniprot.org/align) and pyBoxShade (https://github.com/mdbaron42/pyBoxshade). The structural domains identified from the crystal structure of *T. thermophilus* ClpB (PDB record 1QVR) are indicated with underlines of different colors. The Walker A motifs are highlighted in blue and the Walker B motifs, in yellow.

```
A. phagocytophilum   1 MDLNKFTEKARSFVMQAQVFAISSGHQFLLPEHLLKVMLDEKGSLTENLISMSSCNAGIN
E. chaffeensis       1 MDLNQFTDMSKNLIMQAQTTAIASGHQSLIPEHLLKVMLDTKDELIELLL--TSCGCDID
E. coli.             1 MRLDRLTNKFQLALADAQSLALGHDNQFIEPLHLMSALLNQEGGSVSPLL--TSAGINAG
T. thermophilus      1 MNLERWTQAAREALAQAQVLAQRMKHQAIDLPHLWAVLLKDERSLAWRLL—EKA-GADPK

A. phagocytophilum  61 EIRDALVQHLSKLPVVSGSSSGQLNLSRELAQVLEEASNIAKRNGDAYISAERLLQALVV
E. chaffeensis      59 KIYSDIKLSLSKLPVVSGSGSGHIHLSKEMAQVLEEAISLAKRNQDTYVTVERLLQALAV
E. coli.            59 QLRTDINQALNRLPQVEGTGGDV-QPSQDLVRVLNLCDKLAQKRGDNFISSELFVLAALE
T. thermophilus     59 ALKELQERELARLPKVEGAEVGQ-YLTSRLSGALNRAEGLMEELKDRYVAVDTLVLALAE
                                          N-terminal domain

A. phagocytophilum 121 VN-SNVSRVLINAGVTATKLNTLIEKMREGDKADSETAEQKFDALRKFTQDLTELATEGK
E. chaffeensis     119 VKDTSVYKILLAHGVTPVKLESLILNMRNGSKADTINAEHKFNALKKYAKDITESAMAGK
E. coli.           118 SR-GTLADILKAAGATTANITQAIEQMRGGESVNDQGAEDQRQALKKYTIDLTERAEQGK
T. thermophilus    118 AT-PGLPG---------LEALKGALKELRGGRTVQTEHAESTYNALEQYGIDLTRLAAEGK

A. phagocytophilum 180 MDPVIGRSDETRRLIQVLSRRTKNNPALIGEPGVGKSAIVEGLVQSIVSGNVPIGLQGAK
E. chaffeensis     179 LDPVIGRDEEIRRTMQVLSRRTKNNPVLIGEPGVGKTAIIEGLAQRIVVGDVPVGLRNAK
E. coli.           177 LDPVIGRDEEIRRTIQVLQRRTKNNPVLIGEPGVGKTAIVEGLAQRIINGEVPEGLKGRR
T. thermophilus    169 LDPVIGRDEEIRRVIQILLRRTKNNPVLIGEPGVGKTAIVEGLAQRIVKGDVPEGLKGKR

A. phagocytophilum 240 VLSLDLAALVAGTKYRGEFEERLKAVLSKIILSSGKIILFIDELHMLVGAGSTGDSMDAS
E. chaffeensis     239 IMALDLGMLVAGTKYRGEFEERLKYVINEIVASNGAVILFIDELHTLVGAGATDGAMDAS
E. coli.           237 VLALDMGALVAGAKYRGEFEERLKGVLNDLAKQEGNVILFIDELHTMVGAGKADGAMDAG
T. thermophilus    229 IVSLQMGSLLAGAKYRGEFEERLKAVIQEVVQSQGEVILFIDELHTVVGAGKAEGAVDAG
                               Nucleotide-binding domain 1

A. phagocytophilum 300 NILKPVLARGELRCIGATTLDEYREHIEKDPALARRFQPVFVAEPSINDTISILRGIKEK
E. chaffeensis     299 NLLKPALARGEIHCIGATTLDEYRQHIEKDAALARRFQPVFVSESTVNDTISILRGLKEK
E. coli.           297 NMLKPALARGELHCVGATTLDEYRQYIEKDAALERRFQKVFVAEPSVEDTIAILRGLKER
T. thermophilus    289 NMLKPALARGELRLIGATTLDEYRE-IEKDPALERRFQPVYVDEPTVEETISILRGLKEK

A. phagocytophilum 360 YELHHGIRITDSAIVAAANLSSRYIPDRFLPDKAIDLIDEAASRARIEIDSKPEIIDSID
E. chaffeensis     359 YEVHHGIRIMDSAIIAASTLSNRYITDRFLPDKAIDLIDEAASRVRIEIDSKPEVIDELD
E. coli.           357 YELHHHVQITDPAIVAAATLSHRYIADRQLPDKAIDLIDEAASSIRMQIDSKPEELDRLD
T. thermophilus    348 YEVHHGVRISDSAIIAAATLSHRYITERRLPDKAIDLIDEAAARLRMALESAPEEIDALE

A. phagocytophilum 420 RQVMQLKIESEALKNENTEASKQRLEEISRELQSLSSEAADLNSQWHAEKAKISKMHELT
E. chaffeensis     419 RKIIQLKIEAGVLEKENTESSKQRLAQLSEELNKLSIQATELNSKWQAEKMKILKMQECV
E. coli.           417 RRIIQLKLEQQALMKESDEASKKRLDMLNEELSDKERQYSELEEEWKAEKASLSGTQTIK
T. thermophilus    408 RKKLQLEIEREALKKEKDPDSQERLKAIEAEIAKLTEEIAKLRAEWEREREILRKLREAQ
                                          Middle domain

A. phagocytophilum 480 ESLDSARIELEQSQRLGNLSRAGELMYGIIPSLEAELKKHEEI---AGTLLRKEIKANDI
E. chaffeensis     479 EKLDNARNDLEKAQRSGNLAKAGELMYGIIPELEKELKKCEKP---SSNMLKREVTESDI
E. coli.           477 AELEQAKIAIEQARRVGDLARMSELQYGKIPELEKQLEAATQLEGKTMRLLRNKVTDAEI
T. thermophilus    468 HRLDEVRREIELAERQYDLNRAAELRYGELPKLEAEVEALSEK-LRGARFVRLEVTEEDI
```

```
A. phagocytophilum 537 AAIVERWTGIPVDSVMNSEKEKLLHMEEELKKTVIGQDSAVAAVSNAVRRSRAGVQDAQR
E. chaffeensis     536 ASIVSRWTGIPIENMMSSEKEKLLRMEEEIGKTVIGQESAIKAVSDAVRRSRAGVQDANK
E. coli.           537 AEVLARWTGIPVSRMMESEREKLLRMEQELHHRVIGQNEAVDAVSNAIRRSRAGLADPNR
T. thermophilus    527 AEIVSRWTGIPVSKLLEGEREKLLRLEEELHKRVVGQDEAIRAVADAIRRARAGLKDPNR

A. phagocytophilum 597 PMGSFLFLGPTGVGKTELTKALSKFLFDSSSALLRFDMSEFMEKHSVAKLIGAPPGYVGY
E. chaffeensis     596 PLGSFLFLGPTGVGKTELVKTLAEFLFCDKSALLRFDMSEFMEKHAVSRLIGAPPGYVGY
E. coli.           597 PIGSFLFLGPTGVGKTELCKALANFMFDSDEAMVRIDMSEFMEKHSVSRLVGAPPGYVGY
T. thermophilus    587 PIGSFLFLGPTGVGKTELAKTLAATLFDTEEAMIRIDMTEYMEKHAVSRLIGAPPGYVGY

A. phagocytophilum 657 EQGGLLTEAVRRRPYQVILFDEIEKAHADIFNLLLQVLDEGRLTDSRGNLVNFKNTILVL
E. chaffeensis     656 DQGGMLTESVRRRPYQVILFDEIEKAHGDIFNILLQVLDEGRLTDNHGKLVDFRNTILVL
E. coli.           657 EEGGYLTEAVRRRPYSVILLDEVEKAHPDVFNILLQVLDDGRLTDGQGRTVDFRNTVVIM
T. thermophilus    647 EEGGQLTEAVRRRPYSVILFDEIEKAHPDVFNILLQILDDGRLTDSHGRTVDFRNTVIIL
                                       Nucleotide-binding domain 2

A. phagocytophilum 717 TSNIGQDILINSTE-DSNDPVVRKTVLEMLRLSFRPEFLNRLDEIMIFNRLTQEHIEHIV
E. chaffeensis     716 TSNLGQEILINNKE-DVDGESVKKSITSVLQHHFRPEFLNRLDEIIVFHRLTKEHIEKII
E. coli.           717 TSNLGSDLIQERFG-ELDYAHMKELVLGVVSHNFRPEFINRIDEVVVFHPLGEQHIASIA
T. thermophilus    707 TSNLGSPLILEGLQKGWPYERIRDEVFKVLQQHFRPEFLNRLDEIVVFRPLTKEQIRQIV

A. phagocytophilum 776 DVQISNLQKIISDKGITISLHQGAKSWLVKHGYDVACGARLLKRLIQQHIQNQLACLLLG
E. chaffeensis     775 DVQFSLLQKIVAQKELEISLSSEAKSWLMNNGYDSLYGARPLKRLIQQKIQNSLAKLILA
E. coli.           776 QIQLKRLYKRLEERGYEIHISDEALKLLSENGYDPVYGARPLKRAIQQQIENPLAQQILS
T. thermophilus    767 EIQLSYLRARLAEKRISLELTEAAKDFLAERGYDPVFGARPLRRVIQRELETPLAQKILA

A. phagocytophilum 836 DKITEGSKLVVFEENNSLVIKEAT----
E. chaffeensis     835 NQVSKGDKLEVVLLNDDLIINKL-----
E. coli.           836 GELVPGKVIRLEVNEDRIVAVQ------
T. thermophilus    827 GEVKEGDRVQVDVGPAGLVFAVPARVEA
```

Supplementary Figure 2

Sequence alignment of DnaK from *Anaplasma phagocytophilum*, *Ehrlichia chaffeensis*, *Escherichia coli*, and *Thermus thermophilus*. The alignment was produced with ClustalOmega (https://www.uniprot.org/align) and pyBoxShade (https://github.com/mdbaron42/pyBoxshade). The structural domains are indicated with underlines of different colors.

```
A. phagocytophilum   1 MAAERIIGIDLGTTNSCVAVMEAGTAKVIENSEGSRTTPSVVAFT-DNERLVGELAKRQA
E. chaffeensis       1 ---MAVIGIDLGTTNSCVAVMEGGDAKAIENSEGARTTPSIVAFT-DSERLVGDPAKRQA
E. coli              1 --MGKIIGIDLGTTNSCVAIMDGTTPRVLENAEGDRTTPSIIAYTQDGETLVGQPAKRQA
T. thermophilus      1 --MAKAVGIDLGTTNSVIAVLEGGKPVVLENAEGERVTPSVVAFR-DGETLVGRMAKRQA

A. phagocytophilum  60 NINAQNTIYASKRIIGRRYDDMRDL----KCPYEVFPAKNGDAWIRAKGEGYSPVQIGAF
E. chaffeensis      57 TTNAKNTIYASKRLIGRRYQDVKDI----KSSYDVVSAKNGDAWIKVLGKEYSPSQIGAF
E. coli             59 VTNPQNTLFAIKRLIGRRFQDEEVQRDVSIMPFKIIAADNGDAWVEVKGQKMAPPQISAE
T. thermophilus     58 VLNPEGTIFEIKRFIGRRFEEVQE--EAKRVPYKVVPGPDGGVRVEVKGKLYTPEEISAM

A. phagocytophilum 116 VLEKIKETAERYFGAPVKKAVITVPAYFNDAQRQATKDAGTIAGLDVVRIINEPTAAALA
E. chaffeensis     113 VLEKMKETAERHLGHKVEKAVITVPAYFNDAQRQATKDAGRIAGLDVIRIINEPTAAALA
E. coli            119 VLKKMKKTAEDYLGEPVTEAVITVPAYFNDAQRQATKDAGRIAGLEVKRIINEPTAAALA
T. thermophilus    116 ILRKLVEDASKKLGEKITKAVITVPAYFNNAQREATANAGRIAGLEVLRIINEPTAAALA

A. phagocytophilum 176 YGLDKGDKQRTIVVYDLGGGTFDVSVLEIA----DGVFEVKATNGDTKLGGEDFDNAIME
E. chaffeensis     173 YGLNKSDKQKVIAVYDLGGGTFDVSILEIA----DGVFEVKATNGDTMLGGEDFDHAIMN
E. coli            179 YGLDKGTGNRTIAVYDLGGGTFDISIIEIDEVDGEKTFEVLATNGDTHLGGEDFDSRLIN
T. thermophilus    176 YGLDKK-GNETVLVFDLGGGTFDVTILEIG----EGVFEVKATSGDTHLGGSDMDHAIVN
                                              Nucleotide-binding domain

A. phagocytophilum 232 HMMESFQKETGINLRNDPMAVQRVKEAAEKAKIELSTRLETDITLPFISSDS--TGAKHL
E. chaffeensis     229 YLMDDFKKTTGIDLHNDSMAVQRIKEASEKAKIELSNRMETDINLPFISSDS--TGPKHL
E. coli            239 YLVEEFKKDQGIDLRNDPLAMQRLKEAAEKAKIELSSAQQTDVNLPYITADA--TGPKHM
T. thermophilus    231 WLAEEFKKEHGVDLKADRQALQRLIEAAEKAKIELSSTLETTISLPFIALDPASKTPLHL

A. phagocytophilum 290 SLKLSRAKFEGLVDELIERTIEPCKKALSDAGIKDNSKVDEVVLVGGMTRVPKVIQRVKD
E. chaffeensis     287 SLKLTRAKFENLVDDLIQRTIEPCKKALKDAGISA-DKIDEVVLVGGMTRVPKVIQKVKE
E. coli            297 NIKVTRAKLESLVEDLVNRSIEPLKVALQDAGLSV-SDIDDVILVGGQTRMPMVQKKVAE
T. thermophilus    291 EKKLTRAKFEELIQPLLKRLRGPVEQALKDAGLTP-AQIDEVILVGGATRVPAVQQVVRE

A. phagocytophilum 350 FFGKEPCQGVNPDEVVAVGAAIQGGILTGDVRDVLLLDVAPLSLGIETLGGVFTPLIERN
E. chaffeensis     346 FFGREPHKGVNPDEVVAIGAAIQGSILAGDVRDVLLLDVTPLSLGIETLGGVFTPLIERN
E. coli            356 FFGKEPRKDVNPDEAVAIGAAVQGGVLTGDVKDVLLLDVTPLSLGIETMGGVMTTLIAKN
T. thermophilus    350 LLGKEPNRSVNPDEVVAMGAAIQAGVLMGEVRDVVLLDVTPLSLGVETKGGVMTVLIPRN

A. phagocytophilum 410 TTIPTKKSQVFSTAEDGQTAVTIKVYQGERKMAIDNKLLGQFSLEGIPHAPRGVPQIEVT
E. chaffeensis     406 TTIPTKKSQVFSTAEDGQTAVTIKVYQGERKMAADNKLLGQFSLEGIPSAPRGMPQIEVT
E. coli            416 TTIPTKHSQVFSTAEDNQSAVTIHVLQGERKRAADNKSLGQFNLDGINPAPRGMPQIEVT
T. thermophilus    410 TTIPTRKCEIFTTAEHNQTAVEIHVLQGERPMAQDNKSLGRFRLEGIPPMPAGVPQIEVC
                                                Substrate-binding domain

A. phagocytophilum 470 FDIDANGIVHVSAKDKASGKEQTIKIQSSGGLSDEEIKKMVKDAQDRAEDDEKRKKHVEL
E. chaffeensis     466 FDIDANGIVHVSAKDKASGKEQAIKIQSSGGLSDDEIQRMIKEAEQKAGEDEKRKKFIEL
E. coli            476 FDIDADGILHVSAKDKNSGKEQKITIKASSGLNEDEIQKMVRDAEANAEADRKFEELVQT
T. thermophilus    470 FDIDANGILHVTAKERSTGREASITIQNTTTLSEEEIQRIIEEAKRHAEEDRRRREHAEL
```

```
A. phagocytophilum 530 KNSSEGLIHSVEKSLKDYGDKVAGADKSNIESAIKDLRECLNDSNC-STDTLQQKYDALM
E. chaffeensis     526 KNNGENLVHSTEKSLNEYGDKIPNSDRLEIENAIRDVKDGLSSSDMESVDVLQQKVDHLM
E. coli            536 RNQGDHLLHSTRKQVEEAGDKLPADDKTAIESALTALETALKGEDKAAIEA---KMQELA
T. thermophilus    530 KNALDSARVQAERVLQERQGAP--EARARLEAAIGKAKELVERD-APD-PELKAATEELL
                                                    Lid domain

A. phagocytophilum 589 NLSMKLGEAAYAANKNDGAGSADQSGSSSGGSDGNPEERVVDSEYQEINKDEDKKNT
E. chaffeensis     586 KVSMKLGEALYGNAANNPSSAEN----STASNNEEEDSKVVDSDYQEIDKKDSK---
E. coli            593 QVSQKLMEIAQQQ-----------TAGADASANNAKDDDVVDAEFEEVKDKK-----
T. thermophilus    586 KAVEE-----YEK--------------GAQAASGKGPDDVIDADYKPAD--------
```

Supplementary Figure 3

SDS-PAGE gels stained with Coomassie blue dye of purified *A. phagocytophilum* ClpB (A) and DnaK (B). Migration positions of the molecular weight markers are indicated.

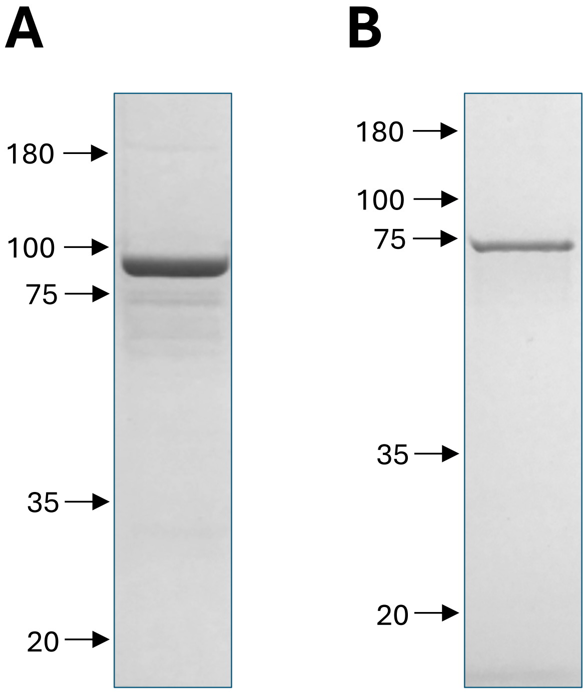

Supplementary Figure 4

Expression of *A. phagocytophilum* ClpB (left panels) and DnaK (right panels) in *E. coli* strains presented in Figures 4, 5 and 6. Top panels: western blot analysis of the cell lysates probed with the polyclonal anti-ClpB and anti-DnaK antisera. Bottom panels: relevant gel fragments stained with Coomassie blue dye. Migration positions of the 100-kDa and 75-kDa markers are indicated. The induction of T7 RNA polymerase (99 kDa) is evident in the presence of IPTG.

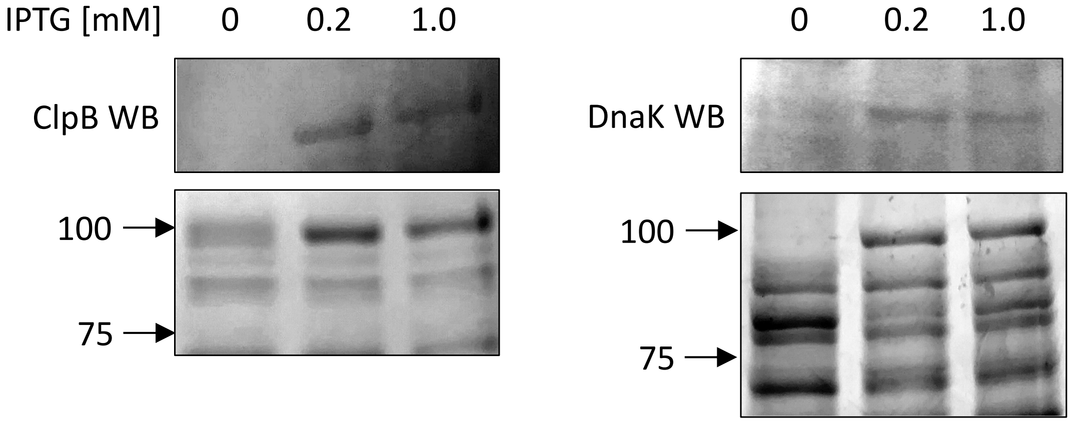

Supplement: Supplementary Fig. 1 — Supplementary material [file mmc1.pdf]
